# Soluble Mediators Produced by Pro-Resolving Macrophages Inhibit Angiogenesis

**DOI:** 10.3389/fimmu.2018.00768

**Published:** 2018-04-25

**Authors:** Shira Michaeli, Vivian Dakwar, Keren Weidenfeld, Ortal Granski, Odelya Gilon, Sagie Schif-Zuck, Anatolii Mamchur, Imad Shams, Dalit Barkan

**Affiliations:** ^1^Department of Human Biology, University of Haifa, Haifa, Israel; ^2^Department of Evolutionary and Environmental Biology, Institute of Evolution, University of Haifa, Haifa, Israel

**Keywords:** pro-resolving macrophages, tissue repair, angiogenesis, antiangiogenic factors, resolution of inflammation, endostatin

## Abstract

Different subtypes of macrophages have been shown to participate in different stages of inflammation and tissue repair. In the late stage of tissue repair, the macrophages, following their engulfment of apoptotic neutrophils, acquire a new phenotype termed alternatively activated macrophages. These macrophages produce growth factors, such as vascular endothelial growth factor (VEGF), that facilitate the angiogenic response as part of tissue restoration. Then, in the later stages of tissue healing, capillary regression takes place. It is presently unknown whether macrophages play an antiangiogenic role in the final stages of tissue repair. Here, we examined whether soluble mediators secreted by pro-resolving CD11b^low^ macrophages (Mres) inhibit angiogenesis in the context of the resolution of tissue repair. Our findings indicate that soluble mediators produced by *ex vivo* generated Mres (CM-Mres) attenuate angiogenesis *in vitro* by inhibiting human umbilical vein endothelial cell (HUVEC) proliferation by lowering their cyclin D1 expression. In addition, CM-Mres lowered HUVEC survival by inducing caspase 3/7 activation, and also inhibited VEGFR2 activation *via* VEGF. HUVEC migration and differentiation to tubular-like structure was also inhibited by CM-Mres. Similarly, CM-Mres significantly inhibited neovascularization as depicted *ex vivo* by utilizing the rat aorta ring assay and *in vivo* by utilizing the chick chorioallantoic membrane assay. Notably endostatin, which was shown previously to exert its antiangiogenic effect by inhibiting proliferation, survival, motility, and morphogenesis of endothelial cells *via* inhibition of VEGFR2 activation, is produced by Mres. Taken together, our results suggest that a specialized subset of macrophages that appear during the resolution of inflammation can produce antiangiogenic mediators, such as endostatin. These mediators can halt angiogenesis, thereby restoring tissue structure.

## Introduction

Inflammation and tissue repair are adaptive responses to tissue damage induced by pathogen infiltration or mechanical or chemical injury. These responses involve sequential stages which are orchestrated by recruitment and activation of various non-hematopoietic and hematopoietic cells such as neutrophils, macrophages, fibroblasts, and endothelial cells (1). The return of the tissue to its homeostatic state is dependent on the tight regulation and final resolution of the inflammatory response and the wound healing processes. However, dysregulated and exaggerated tissue repair that fails to subside and resolve will result in fibrosis and consequently will lead to organ failure (2, 3). Therefore, it is important to understand the contribution of the different cellular mediators in the resolution of inflammation and the various stages of tissue repair.

Macrophages are highly versatile immune cells that can acquire functionally distinct phenotypes (4, 5). Indeed, recent reports suggest the role of specific subpopulations of macrophages in regulating the different stages of tissue repair (6) and resolution of inflammation (7). In the initial stage of the inflammatory response to injury, leukocyte infiltration is followed by recruitment of monocytes to the site of injury. The monocytes differentiate to classically active macrophages also known as M1-like macrophages (pro-inflammatory) (4, 5). The phenotype of M1-like macrophages that engulf apoptotic polymorphonuclear leukocytes cells (PMN) shifts to that of alternatively activated macrophages. The latter are also referred to as M2-like macrophages and are involved in attenuating inflammation and promoting tissue repair (8, 9). Specifically, M2-like macrophages promote tissue repair by secreting growth factors such as transforming growth factor beta-1 which induces myofibroblast differentiation and deposition of extracellular matrix, and vascular endothelial growth factor (VEGF) which promotes angiogenesis (2, 10). Hence, tight temporal regulation of macrophage phenotype is required to promote resolution of inflammation, tissue repair, and reinstatement of homeostasis. The potential role of macrophages in resolving tissue repair and inflammation has been recently described (7, 11). Schif-Zuck et al. characterized a novel subset of pro-resolving macrophages designated CD11b^low^ macrophages that appear during the resolution of zymosan-induced murine peritonitis. These macrophages secrete pro-resolving mediators and are generated *in vivo* and *ex vivo* from M2-like macrophages following the engulfment of apoptotic leukocytes (11). However, these macrophages display a distinct enzyme expression signature from either M1 or M2, are devoid of phagocytic potential, and are prone to migrate to lymphoid tissues. We recently demonstrated that these pro-resolving macrophages can secrete anti-fibrotic mediators, thus preventing the establishment of a fibrotic-like milieu by preventing the expression of type I collagen (Col-I) by activated myofibroblasts (Gilon et al., submitted for publication). Notably, Col-I remodeling and vasculature regression are evident in the late phase of resolution. Furthermore, intraperitoneal zymosan injection is a model of acute inflammation which self-resolves within 48–72 h (12). Hence, we hypothesized that the recently characterized, pro-resolving macrophages secrete antiangiogenic mediators in addition to anti-fibrotic mediators, thus finalizing tissue repair. Here, we demonstrate that *ex vivo* generated pro-resolving CD11b^low^ macrophages (Mres) secrete antiangiogenic mediators such as endostatin thereby inhibiting angiogenesis by endothelial cells.

## Materials and Methods

### Cell Line Cultures

Human umbilical vein endothelial cells (HUVECs) (kindly provided by Prof. Gera Neufeld, Technion, Israel) were grown on 10 cm plates, coated with 0.2% gelatin in Dulbecco’s phosphate-buffered saline (PBS; Biological Industries, Israel) and overlaid with growth medium comprised of Earle’s salt base (M-199) medium supplemented with 20% fetal bovine serum (FBS), 1% antibiotics, 1% vitamins, and glutamine (Biological Industries, USA) and freshly added basic fibroblast growth factor (bFGF) (PeproTech, Israel) (5 ng/ml). Jurkat T cells (kindly provided by Prof. Debbie Yablonski, Technion, Israel) were maintained in RPMI-1640 (Gibco–Life Technology, USA) with high glucose, 10% heat inactivated FBS, and 1% antibiotics. All cells were incubated at 37°C, 5% CO_2_ incubator.

### Animals

7- to 8-week-old male C57BL/6 mice and 6-week-female Sprague Dawley rats were purchased from Harlan Biotech Israel. All animals were maintained under specific pathogen-free conditions. Care and handling of animals was in compliance with University of Haifa’s experimental protocols. This study was carried out in accordance with the recommendations of University of Haifa Animal Ethics Committee guidelines. The protocol was approved by the University of Haifa Animal Ethics Committee.

### *Ex Vivo* Generation of Pro-Resolving CD11b^low^ Macrophages

Male C57BL/6 mice were injected i.p. with zymosan A (1 mg) purchased from Sigma-Aldrich, Israel. After 66 h, peritoneal exudates were collected, and exudate cells were stained with PE-conjugated rat anti-F4/80 (BioLegend Inc., USA). Macrophages were isolated using EasySep PE selection magnetic beads following the manufacturer’s instructions (StemCell Technologies, Israel). Isolated macrophages were co-stained with FITC-conjugated rat anti-Ly-6G and PerCP-conjugated rat anti-mouse CD11b (BioLegend Inc., USA) and analyzed by FACSCanto II (BD Biosciences, USA) and the FACSDiva software. Jurkat T cells were treated with 1 µM staurosporine (Sigma-Aldrich, Israel) to induce apoptosis and washed. Then, peritoneal macrophages were incubated in the presence or absence of apoptotic Jurkat T cells [1:5 macrophage to apoptotic cell (AC) ratio]. After 8 h of incubation, the cells were washed with PBS and overlaid with fresh media; RPMI-1640 with high glucose 10% FBS, and 1% antibiotics for additional 12 h of incubation. Next, conditioned media were collected, and the macrophages were further characterized for their conversion to the CD11b^low^ phenotype using flow cytometry.

### Preparation of the Different Conditioned Media for the Experimental Assays

The following conditioned media, listed below, were diluted with HUVEC Assay Medium (M-199 medium supplemented with 10% FBS, 1% antibiotics, 1% vitamins, 1% glutamine, and freshly added bFGF 5 ng/ml) at a ratio of 1:1. This step was carried out to ensure viability of HUVEC in all the experimental assays described below.

Condition medium (CM): Baseline conditioned media comprise RPMI supplemented with 10% heat inactivated FBS and 1% antibiotics.
CM-Mϕ: Conditioned media collected after 12 h from culture enriched with CD11b^high^ macrophages (Mϕ).
CM-Mres: Conditioned media collected after 12 h from culture enriched with CD11b^low^ macrophages (Mres).
CM-AC: Conditioned media of un-engulfed ACs.

### Proliferation Assay

Human umbilical vein endothelial cell grown in 10 cm plates were treated for 2 h in M-199, 5% FBS, 1% antibiotics, 1% vitamins, and 1% glutamine medium. Next, the above treated cells (3 × 10^3^ cells/well) were cultured in 96-wells plate coated with Cultrex^®^ growth factor-reduced basement membrane extract (BME) (Trevigen Inc., USA) and treated with the different conditioned media. After overnight incubation at 37°C, 5% CO_2_ incubator Cell Titer 96 AqueousOne Solution cell proliferation assay kit (Promega, USA) was added to the wells for 2 h to measure cell proliferation according to the manufacturer’s instructions. The absorbance was recorded at 490 nm.

### Immunofluorescence Staining

Human umbilical vein endothelial cell cultured in 8-well chamber glass slides coated with BME (Trevigen Inc., USA), as described previously (13), were treated for 5 min with mixture containing 0.1% Triton X-100 and 4% PFA containing 5% sucrose, and fixed for an additional 25 min with 4% PFA containing 5% sucrose. The cells were washed for 10 min with PBS and an additional 15 min with PBS containing 0.05% Tween 20 (PBS-T). Next, fixed cells were blocked with IF buffer (130 mM NaCl, 7 mM Na_2_HPO_4_, 3.5 mM NaH_2_PO_4_, 7.7 mM NaN_3_, 0.1% BSA, 0.2% Triton X-100, and 0.05% Tween 20) containing 10% donkey serum for 1 h followed by overnight incubation at 4°C with rabbit anti-active-caspase-3 (1:400) (Cat # 559565; BD Biosciences). The cells were washed three times with PBS for 15 min each and incubated for 1 h with donkey anti-rabbit conjugated to Alexa Fluor^®^ 647 (Invitrogen, USA) at room temperature. Next, the cells were washed as mentioned earlier and mounted with VECTASHIELD mounting medium with 4′,6-diamidino-2-phenylindole (DAPI). Immunofluorescent images were captured by Zeiss LSM 700 confocal laser scanning microscope (magnification 40×).

For F-actin staining, cells were incubated overnight with Alexa Fluor 488 Phalloidin (1:40) (Molecular Probes, USA), washed three times with PBS for 15 min each and mounted with VECTASHIELD mounting medium with DAPI.

### Caspase 3/7 Activity

Human umbilical vein endothelial cells grown in 10 cm plates were treated for 2 h in M-199, 5% FBS, 1% antibiotics, 1% vitamins, and 1% glutamine medium. Next, the aforementioned treated cells were cultured in 96 wells coated with 50 µl BME (3 × 10^3^ cells/well) and were overlaid with the different conditioned media. After overnight incubation at 37°C, 5% CO_2_ incubator, Caspase-Glo^®^ reagent (Promega, USA) was added for each well according to the manufacturer’s instructions, and plates were incubated at room temperature for 1 h. Luminescence of each sample was measured using a plate-reading infinite M200PRO, TECAN luminometer.

### Wound Migration Assay

Wound migration assay was performed using a 12-well plate coated with 0.2% gelatin in PBS (10 × 10^4^ HUVEC/well). 17 h post seeding, a wound was induced by mechanical application of a 1,000 µl sterile tip. Images of wound formation and healing were acquired at time 0 and at 2.5, 5.5, 7, and 8.5 h post-induction, using a light inverted microscope magnification 10× (Nikon Eclipse TS100). Analysis of the wound healing was carried using the Nikon A1R confocal laser scanning microscopy software (NIS Elements AR version 4.3, by Nikon). The area of the scratch was quantified and normalized to the area of the scratch at time 0.

### Time-Lapse Microscopy

Human umbilical vein endothelial cells (6 × 10^4^) were plated on top of gelatinized 15 mm glass-bottom cell culture dishes (Nest Scientific USA Inc.) and overlaid with either CM-Mϕ or CM-Mres. Cells were incubated at 37°C, 5% CO_2_ incubator for 30 min to allow adherence of the cells. Thereafter, cell motility was followed by time-lapse video microscopy using Nikon A1R confocal laser scanning microscope (20× magnification). Differential interference contrast (DIC) microscopy images were acquired every 1 min for a period of 2 h. Motility of the cells for a period of 2 h was measured by determining the average velocity of 10 different cells for each treatment utilizing ImageJ software (with the “win 64” plug).

### Tube-Formation Assay

Human umbilical vein endothelial cells grown in 10 cm plates were treated for 2 h in M-199, 5% FBS, 1% antibiotics, 1% vitamins, and 1% glutamine medium. These cells were then cultured in eight-chamber glass slides (Lab-TEK^®^ II, Naperville, IL, USA) coated with BME and overlaid with the different conditioned media for 16 h. For positive control M-199, 20% FBS, 1% antibiotics, 1% vitamins, and 1% glutamine medium was applied. Pictures were acquired by Nikon Eclipse TS100 light microscopy (10× magnification), and the number of bifurcations per field was quantified using ImageJ software.

### Aortic Ring Assay

Aorta ring assay was carried out as previously described (14) with slight modification. Briefly, thoracic aorta rings were prepared from female Sprague Dawley rats according to the protocol by Bellacen and Lewis (14) and placed in 48-well plates coated with 150 µl Cultrex^®^ growth factor-reduced BME. Plates were incubated for 10 min at 37°C in a 5% CO_2_ incubator. Following incubation, each well was overlaid with an additional 150 µl BME and incubated at 37°C, in a 5% CO_2_ incubator for 20–30 min. Next, the aorta rings were subjected to the different treatments for a period of 6 days. For a negative control, vascular cell basal medium (ATCC^®^ PCS-100-030™) was applied, and for positive control vascular cell basal medium supplemented with components listed in Table S1 in Supplementary Material was utilized. Images were acquired by Stemi SV 6, ZEISS light microscope (10× magnification). The micro-vessel sprouting area was analyzed by ImageJ software. Sprouting area for each treatment at time point *T* = 0 was subtracted from the sprouting area at 2, 4, and 6 days of treatment.

### Chick Chorioallantoic Membrane (CAM) Assay

The CAM assay was carried out as previously described (15) with slight modification. Embryonated chicken eggs (~30 per treatment) were incubated at 38°C incubator. At day 3, ovalbumin was removed (3 ml per egg), a window was opened [according to the protocol of Ponce and Kleinmann (15)], and inserts treated with the different conditioned media were applied. The inserts were composed of autoclaved filtered paper (5.5 mm in diameter), which were treated either with CM-Mϕ or CM-Mres (10 µl/insert). Eggs were incubated for additional 48 h at 38°C incubator. Images were acquired by binocular (0.8× magnification) at time 0 and after 48 h incubation with the above inserts. Quantification of blood vessel density was carried out using ImageJ (by using “win 64” plug). For each treatment, an area of 15 cm^2^ was analyzed with the filter paper at its center. Vascular density measured at 48 h posttreatment was normalized to the vascular density measured at time 0.

### Mouse Angiogenesis Array

Mouse angiogenesis array kit (R&D Biosystems) was used according to the manufacture’s instructions using either CM-Mϕ or CM-Mres. Membranes were analyzed using ImageQuant LAS-4000 analyzer (GE Healthcare Life Sciences, Pittsburgh, PA, USA) and “ImageQuant LAS-4000” software (GE Healthcare Life Sciences). Densitometry analysis was performed using ImageQuant total lab-7 (GE Healthcare Life Sciences) image analysis software.

### VEGFR2 Phosphorylation

Confluent HUVEC cells (cultured on gelatin as described earlier) were treated in M-199, 5% FBS, 1% antibiotics, 1% vitamins, and 1% glutamine medium for 12 h. Next, the cells were overlaid with the different conditioned media for 30 min followed by VEGF supplementation (25 ng/ml) for 5 min. Cell pellets were prepared for western blot analysis.

### Western Blot Analysis

Cell pellets were lysed in WCE (whole-cell extract) buffer [25 mM HEPES, pH 7.7, 0.3 M NaCl, 1.5 mM MgCl_2_, 0.2 mM EDTA, 0.1% Triton X-100, 100 µg/ml PMSF, and 25 mM protease inhibitor cocktail (Roche)], and for p-VEGFR2 detection the cell pellets were lysed in WCE buffer supplemented with 20 mM NAF, 2 mM Na_3_VO_4_, and 0.5 mM DTT. The proteins from cell lysate or from the conditioned media were separated by SDS-PAGE followed by transfer on to a nitrocellulose membrane. The membranes were blocked with 5% (w/v) non-fat dried skimmed milk powder either in PBS supplemented with 0.05% Tween 20 (PBS-T) for protein detection or for phosphorylated VEGFR2 in Tris-buffered saline supplemented with 0.1% Tween 20 (TBS-T) for 1 h at room temperature. Membrane was then probed either with rabbit anti-GAPDH (1:500), rabbit anti-cyclin D1 (1:200), or rabbit anti-VEGFR2 (1:1,000) (Santa Cruz, Dallas, TX, USA). Rabbit anti-phospho-VEGFR2-Tyr951 (1:500) (Cell Signaling, Danvers, MA, USA), rabbit anti-VEGF (Abcam, Cambridge, UK), or monoclonal anti-endostatin (1:1,000) (Merck, Darmstadt, Germany) at 4°C overnight. Next, the membranes were incubated with the appropriate horseradish peroxidase-conjugated secondary antibodies (1:10,000; Jackson ImmunoResearch Laboratories, West Grove, PA, USA) for 1 h at room temperature and washed 15 min 3× with either PBS-T or TBS-T (for phosphor-protein detection). Western Bright ECL (Advansta, Menlo Park, CA, USA) was added to the membrane for 1 min and analyzed using ImageQuant LAS-4000 analyzer (GE Healthcare Life Sciences, Pittsburgh, PA, USA) and “ImageQuant LAS-4000” software (GE Healthcare Life Sciences). Densitometry analysis was performed using ImageQuant total lab-7 (GE Healthcare Life Sciences) image analysis software.

### Statistical Analysis

Student’s unpaired *t*-test was used accordingly. Two-tailed *p* values of 0.05 or less were considered to be statistically significant. Repeated measures ANOVA comparison test was used accordingly. Values of 0.05 or less were considered to be statistically significant.

## Results

### *Ex Vivo* Generation of Secreted Factors of Pro-Resolving CD11b^low^ Macrophages

To this end, peritonitis was induced, and 66 h later peritoneal exudates were collected. The percentage of macrophages (Mϕ) was determined in peritoneal exudates, based on their size and granularity and positive staining for F4/80 as previously described (11). Next, CD11b^high^ Mϕ were collected and were either untreated or treated with apoptotic Jurkat cells {a common apoptotic leukocyte target for macrophages in experimental procedures [(11, 16) at a ratio of 1:5 respectively]}. Incubation with ACs resulted in 79% conversion of CD11b^high^-Mϕ to CD11b^low^-Mϕ compared with untreated macrophages where only 13% of CD11b^high^-Mϕ were converted to CD11b^low^-Mϕ, as determined by surface expression of CD11b by FACS analysis (Figure S1 in Supplementary Material). Conditioned media were collected after 12 h from cultures enriched with either CD11b^high^ macrophages (CM-Mϕ) or pro-resolving CD11b^low^ macrophages (CM-Mres) and from un-engulfed AC (CM-AC).

### Proliferation of HUVECs is Modulated by Factors Secreted by Pro-Resolving CD11b^low^ Macrophages

**Figure 1:**
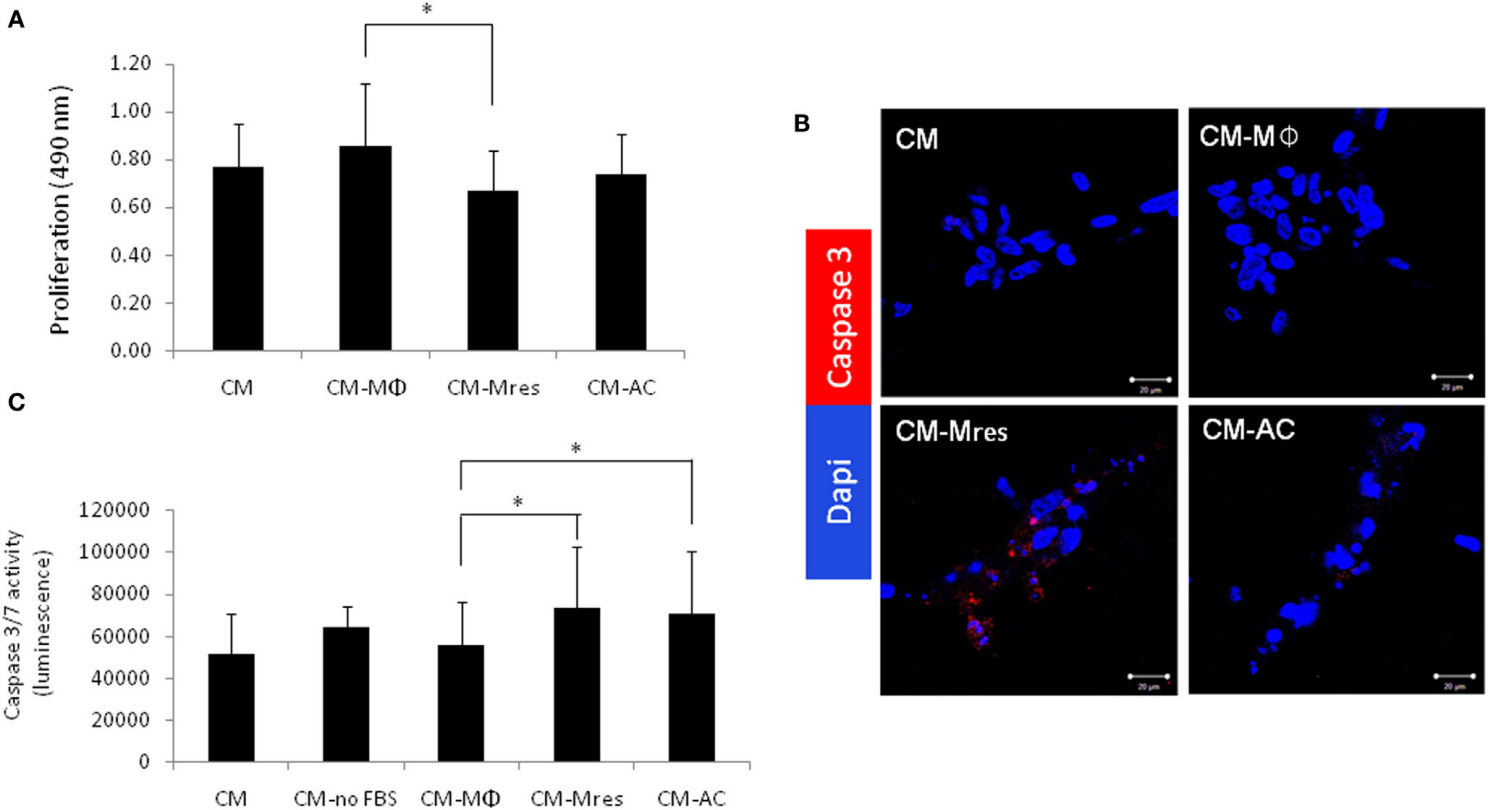
CM-Mres inhibits proliferation and induces apoptosis of human umbilical vein endothelial cell (HUVEC). HUVEC were cultured on basement membrane extract and treated with condition medium (CM), CM-Mϕ, CM-Mres, or CM-AC. **(A)** Representative proliferation of HUVEC after overnight treatment. *n* = 5 with four to five replicates. **(B)** Representative confocal images of HUVEC stained for activated caspase 3 (red) and nuclei (DAPI, blue). Magnification 40×, bar = 50 µm. **(C)** Caspase 3/7 activity in HUVEC either starved [CM-no-fetal bovine serum (FBS); positive control] or treated with the indicated conditioned media overnight. Columns; mean, bars; STD, *n* = 6 with three replicates for each experiment. One-way repeated measures ANOVA analysis with repeated contrasts, *p ≤ 0.05.

Angiogenesis, characterized by sprouting of preexisting vasculature to form new vessels, requires several coordinated endothelial cell activities, such as proliferation, migration, and morphogenesis (17, 18). Therefore, initially we determined whether CM-Mres was able to inhibit the proliferation of endothelial cells. To this end, conditioned media (condition medium (CM), CM-Mϕ, CM-Mres, or CM-AC) were overlaid on HUVECs cultured on top of growth factor-reduced reconstituted BME. The proliferation was measured after overnight incubation. One-way repeated measures ANOVA analysis was conducted followed with repeated contrasts to probe the differences between the four groups of treatments. Significant difference was found between the different treatments [*F*(3, 12) = 5.699, *p* = 0.012]. Furthermore, significant difference was found between the treatments CM-Mϕ vs. CM-Mres (*p* = 0.022) (Figure 1A). By contrast, no significant difference was found between CM-Mϕ vs. CM-AC (*p* > 0.05). Given that we observed a reduction in cell number of HUVEC treated with CM-Mres, we next tested whether this inhibition is also due to an increase in apoptosis of HUVEC. HUVECs were cultured as described earlier and scored for apoptosis by (1) percentage of TUNEL positive cells (Figure S2 in Supplementary Material), (2) activated caspase 3 detected by immunofluorescence staining (Figure 1B), and caspase 3/7 activity (Figure 1C). Increase in the percentage of HUVEC positive for TUNEL staining was evident upon treatment with CM-Mres compared with CM-Mϕ (Figure S2 in Supplementary Material). Activated caspase 3 was apparent by immunofluorescence staining (red staining) in HUVEC treated with CM-Mres (Figure 1B), whereas no staining was evident in CM-Mϕ. In addition, a significant increase in caspase 3/7 activity was observed in HUVEC treated either with CM-Mres or CM-AC compared with CM-Mϕ as determined by one-way repeated measures ANOVA analysis followed with repeated contrasts. Specifically, significant difference was found between the different treatments [*F*(4, 20) = 4.549, *p* = 0.009]. Furthermore, significant difference was found between CM-Mres compared with CM-Mϕ (*p* = 0.014) (Figure 1C) and CM-AC compared with CM-Mϕ (*p* = 0.030). Hence, our results suggest that CM-Mres inhibits proliferation. Of note, activation of caspase 3/7 by CM-Mres may be also attributed to the presence of mediators secreted by un-engulfed ACs.

### CM-Mres Inhibits the Motility of HUVEC

**Figure 2:**
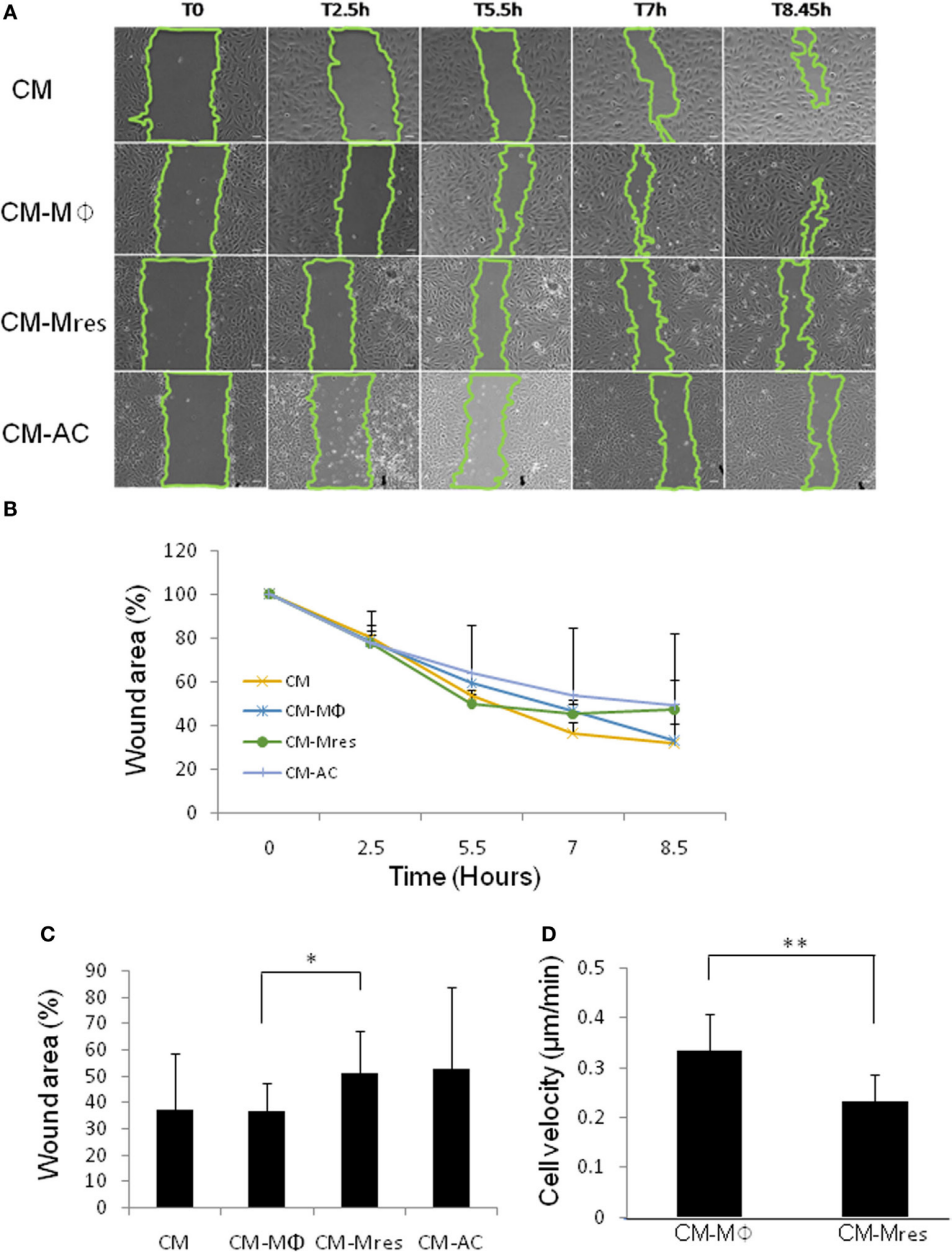
Motility of human umbilical vein endothelial cell (HUVEC) is attenuated upon treatment with CM-Mres. **(A,B)** Representative results (*n* = 4) of wound migration assay of HUVEC treated with the indicated conditioned media. **(A)** Images taken at indicated time points demonstrate quicker wound closure upon treatment with either condition medium (CM) or CM-Mϕ compared with treatment with CM-Mres or CM-AC. (B) Quantification of the wound closure (motility of the cells) over time. Points; mean, bars; STD, *n* = 4. **(C)** Quantification of the wound closure at 8.5 h post wounding. Columns; mean, bars; STD, *n* = 6, one-way repeated measures ANOVA analysis with repeated contrasts, *p ≤ 0.05. **(D)** Motility of HUVEC cultured on gelatin for a period of 2 h upon treatment with either CM-Mϕ or CM-Mres was monitored by time-lapse microscopy. An average velocity of 10 different cells for each treatment was determined. Columns; mean, bars; STD, *n* = 10 cells for each treatment, *t*-test, *p ≤ 0.05, **p ≤ 0.01.

Migration of vascular endothelial cells plays an important role in angiogenesis (18). Therefore, we tested whether CM-Mres may impact the motility of HUVEC. To this end, wound migration assay was utilized to study the rate of the wound closure in the plate (cell migration toward the wound/scratch) as detailed below. Wounded monolayers of HUVECs were incubated with the different conditioned media (CM, CM-Mϕ, CM-Mres, or CM-AC). The filling of the “wound” was monitored in a period of 2.5–8.5 h by measuring the % of remaining clear surface, compared with the *T* = 0 (scratch initiation). Our results demonstrate that treatment with CM-Mres delayed overtime the closure of the wound (Figures 2A,B) compared with treatment with CM-Mϕ. Repeated measures ANOVA and repeated contrasts were conducted with treatment (CM, CM-Mϕ, CM-Mres, and CM-AC) as repeated measures, at time 8.5 h. A significant difference between all treatments was found [*F*(3, 3) = 24.261, *p* = 0.013].

In addition, at 8.5 h posttreatment, CM-Mres significantly inhibited the closure of the wound compared with CM-Mϕ as determined by repeated contrast [*F*(1, 5) = 7.548, *p* = 0.040]. Specifically, 51% of the original area remained (Figure 2C) upon treatment with CM-Mres whereas, 36% of the original area remained upon treatment with CM-Mϕ (Figure 2C). Whereas, there was no significant difference between CM-Mϕ compared with CM-AC (*p* > 0.05).

To further validate that the delay in wound closure was due to inhibition of motility of HUVEC, we conducted time-lapse video microcopy for a period of 2 h (DIC images were captured every minute). Indeed, CM-Mres treatment significantly attenuated the motility of HUVEC cultured on gelatin (Video S1 in Supplementary Material; Figure 2D) compared with treatment with CM-Mϕ (Video S2 in Supplementary Material; Figure 2D). Interestingly, CM-Mϕ altered HUVEC morphology and induced cell expansion and contraction often associated with membrane blebbing upon their movement, whereas treatment with CM-Mres induced a spindle shape morphology and no cell expansion or membrane blebbing was apparent in the migrating HUVEC.

In summary, our findings suggest that CM-Mres inhibits the motility of HUVEC.

### Soluble Mediators Secreted by Pro-Resolving Macrophages Prevent HUVEC Morphogenesis to Tubular Structures

**Figure 3:**
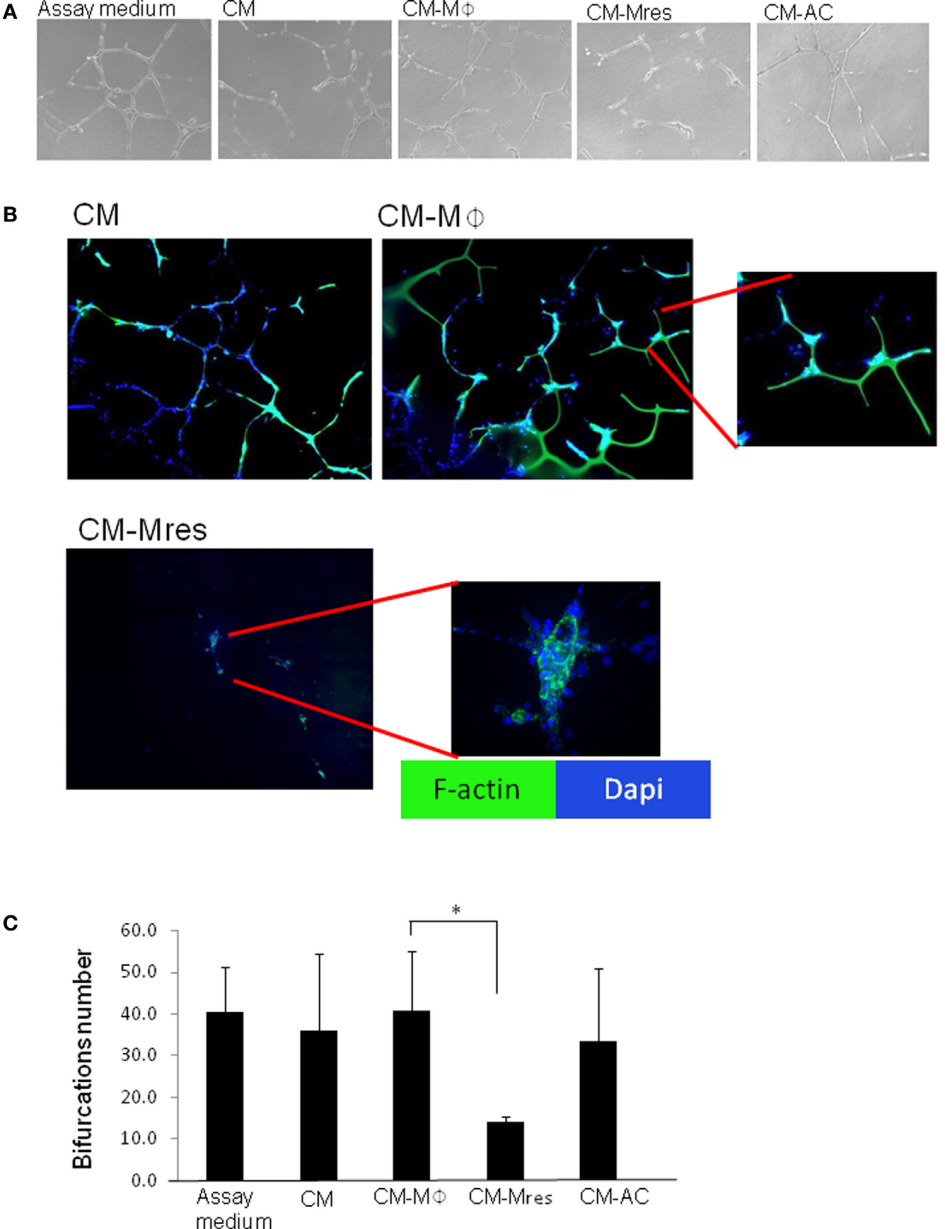
CM-Mres hinders human umbilical vein endothelial cell (HUVEC) differentiation to tubular-like structures. HUVECs were cultured on basement membrane extract for 16–19 h with Assay Medium, condition medium (CM), CM-Mϕ, CM-Mres, or CM-AC. **(A)** Representative light microscopy images (*n* = 5). Magnification 10×. **(B)** Fluorescence staining of HUVEC cells for F-actin (phalloidin; green) and nuclei (DAPI; blue). Representative confocal images are shown. Magnification 40×. Bars = 50 μm. **(C)** Quantification of the bifurcation number of vessel-like tubular structures obtained from three to five microscopic fields. Columns; mean, bars; STD, *n* = 5, one-way repeated measures ANOVA analysis with repeated contrasts, *p < 0.05.

Next, we determined whether soluble mediators secreted by pro-resolving macrophages were able to inhibit the differentiation of endothelial cells to capillary-like networks. To this end, HUVECs were cultured on BME and treated with CM, CM-Mϕ, CM-Mres, CM-AC, or Assay Medium that promotes HUVEC differentiation to tubular structures. After 16–18 h, the extent of HUVEC differentiation to tubular structures was determined by light microscopy (Figure 3A). Quantification of the number of bifurcations of vessel-like tubular structures was carried out in three independent fields per each experimental condition using ImageJ software (Figures 3A,C). F-actin organization of the tubular-like structures was determined by phalloidin staining (Figure 3B).

HUVEC grown on BME with Assay Medium undergo spontaneous alignment into hollow tubes, forming capillary-like networks (19). However, we found that endothelial tubule formation and stability was impaired in the presence of CM-Mres compared with endothelial cells treated with CM, Assay Medium, or CM-Mϕ (Figures 3A,B). This was further supported by one-way repeated measures ANOVA analysis demonstrating significant differences between the different treatments [*F*(4, 16) = 9.997, *p* < 0.001]. Utilizing repeated contrasts analysis, we found a significant reduction in the number of bifurcations in the tubular network formed by the endothelial cell treated with CM-Mres in comparison to treatment with CM-Mϕ (*p* = 0.012) (Figure 3C). By contrast, there was no significant difference between CM-AC in comparison to CM-Mϕ (*p* > 0.05). The tube-formation assay is based on the ability of endothelial cells to form three-dimensional capillary-like tubular structures in the 3D BME system. In this system, endothelial cells proliferate, differentiate, directionally migrate to align, branch, and form the tubular polygonal networks of blood vessels. This is a well-established assay to study angiogenesis (19, 20). Given that a significant effect on formation of the capillary-like tubular structures was only evident upon treatment with CM-Mres, we proceeded to further validate the antiangiogenic effect of CM-Mres and compared it to CM-Mϕ, as described below.

### Sprouting Angiogenesis *Ex Vivo* and *In Vivo* are Attenuated by CM-Mres

**Figure 4:**
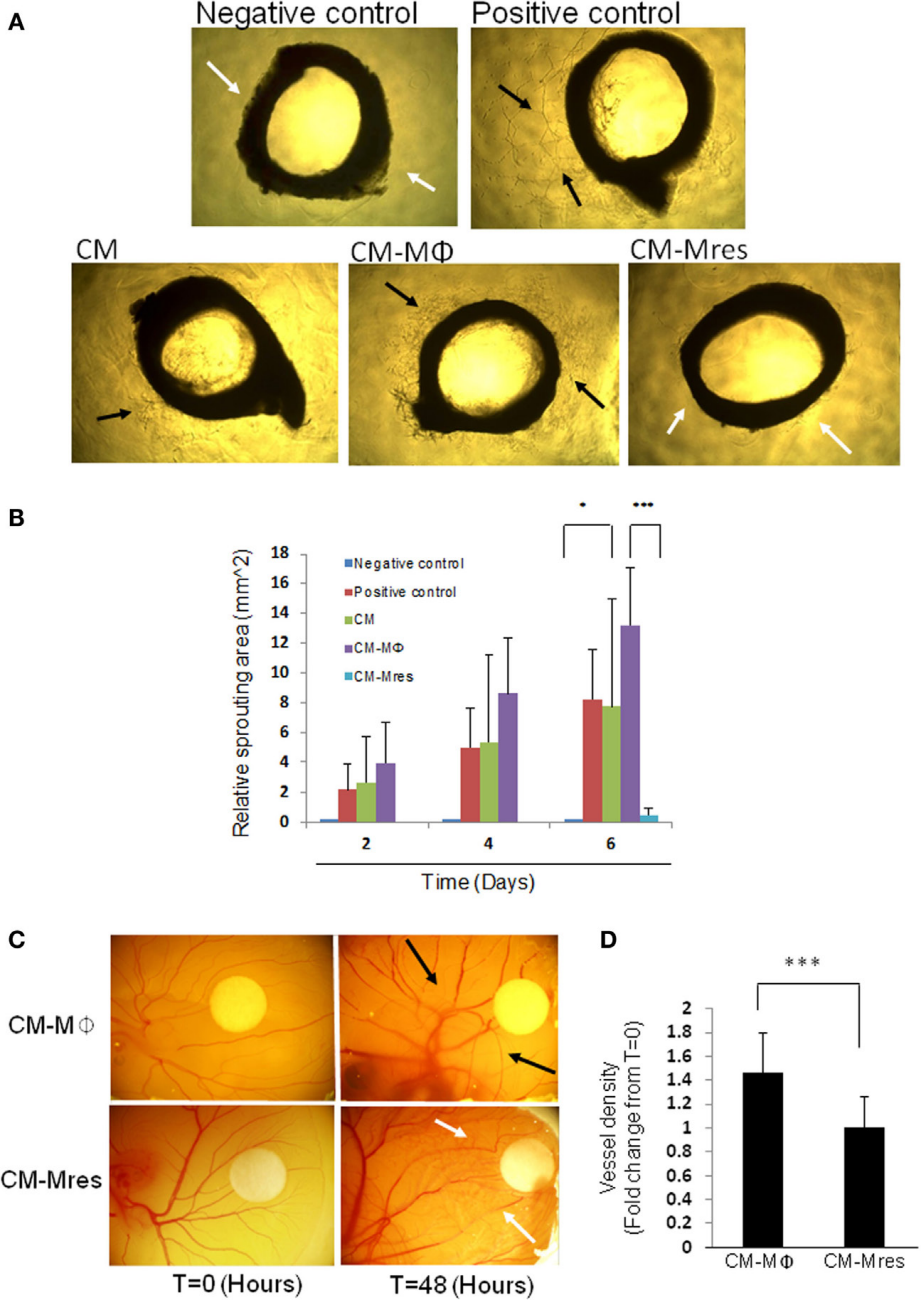
Effect of CM-Mres on angiogenesis of rat aorta ring and chick chorioallantoic membrane (CAM). **(A)** Rat aortic ring assay. Representative photomicrographs of rat aortic ring sections from 6-week-old rat cultured on basement membrane extract and treated for 6 days with basal media (negative control), basal media with supplementations (positive control, see Materials and Methods), condition medium (CM), CM-Mϕ, or CM-Mres. Vascularized area is indicated by black arrow heads, and avascular area is indicated by white arrow heads. **(B)** Quantification of the sprouting area of endothelial cells relative to *T* = 0. Columns; mean, bars; STD, *n* = 2 with three replicates for each treatment, two-way repeated measures ANOVA analysis with repeated contrasts *p ≤ 0.05, ***p ≤ 0.001. **(C)** Representative images of the chick CAM treated for 48 h with either CM-Mϕ (*n* = 15) or CM-Mres (*n* = 12). Vascularized area is indicated by black arrow heads, and avascular area is indicated by white arrow head. **(D)** Quantification of vessel density in panel **(C)**. Columns; mean of the fold change in vessel density from *t* = 0 for each treatment, bars; STD, *t*-test, ***p ≤ 0.001.

To further verify our *in vitro* findings, we utilized the rat aorta ring assay as an *ex vivo* model of angiogenesis. This organ culture assay scores for sprouting angiogenesis from the segmented aorta ring cultured on BME. Our results demonstrate that CM-Mϕ promoted neovascularization (see black arrow; Figure 4A; Figure S3 in Supplementary Material), whereas, CM-Mres restrained neovascularization (see white arrows; Figure 4A). We conducted repeated measures two-way ANOVA analysis, and we found significant difference between the different treatments [*F*(4, 40) = 10.828,*p* = 0.003]. Furthermore, we found significant interactions between time and treatment [*F*(8, 40) = 14.127, *p* < 0.001]. Planned comparisons were carried out on data from day 6 to probe the interaction. We found significant difference between CM-Mres compared with CM-Mϕ [*F*(1, 5) = 55.574, *p* = 0.001]. Furthermore, a significant difference was found between negative control vs. CM [*F*(1, 5) = 6.663, *p* = 0.049]. Whereas, no significant difference was found between CM-Mres vs. negative control [*F*(1, 5) = 4.839, *p* = 0.079] (Figure 4B). Hence, CM-Mres contain soluble mediators that can inhibit sprouting angiogenesis in the rat aorta ring assay. Next, we tested whether CM-Mres can inhibit angiogenesis *in vivo* by using the chick CAM assay. Similarly, to the *in vitro* and *ex vivo* results, exposure of the CAM to CM-Mres reduced vessel density (compare vessel density at time *T* = 0 vs. *T* = 48 h post CM-Mres treatment; Figure 4C, see white arrows and Figure 4D) whereas, treatment with CM-Mϕ for 48 h increased vessel density compared with *T* = 0 (Figure 4C see black arrows and Figure 4D). Altogether, these results suggest that CM-Mres contains antiangiogenic mediators.

### Increased Levels of the Antiangiogenic Mediator Endostatin and Decrease in Angiogenic Factor VEGF in CM-Mres Compared with CM-Mϕ

**Figure 5:**
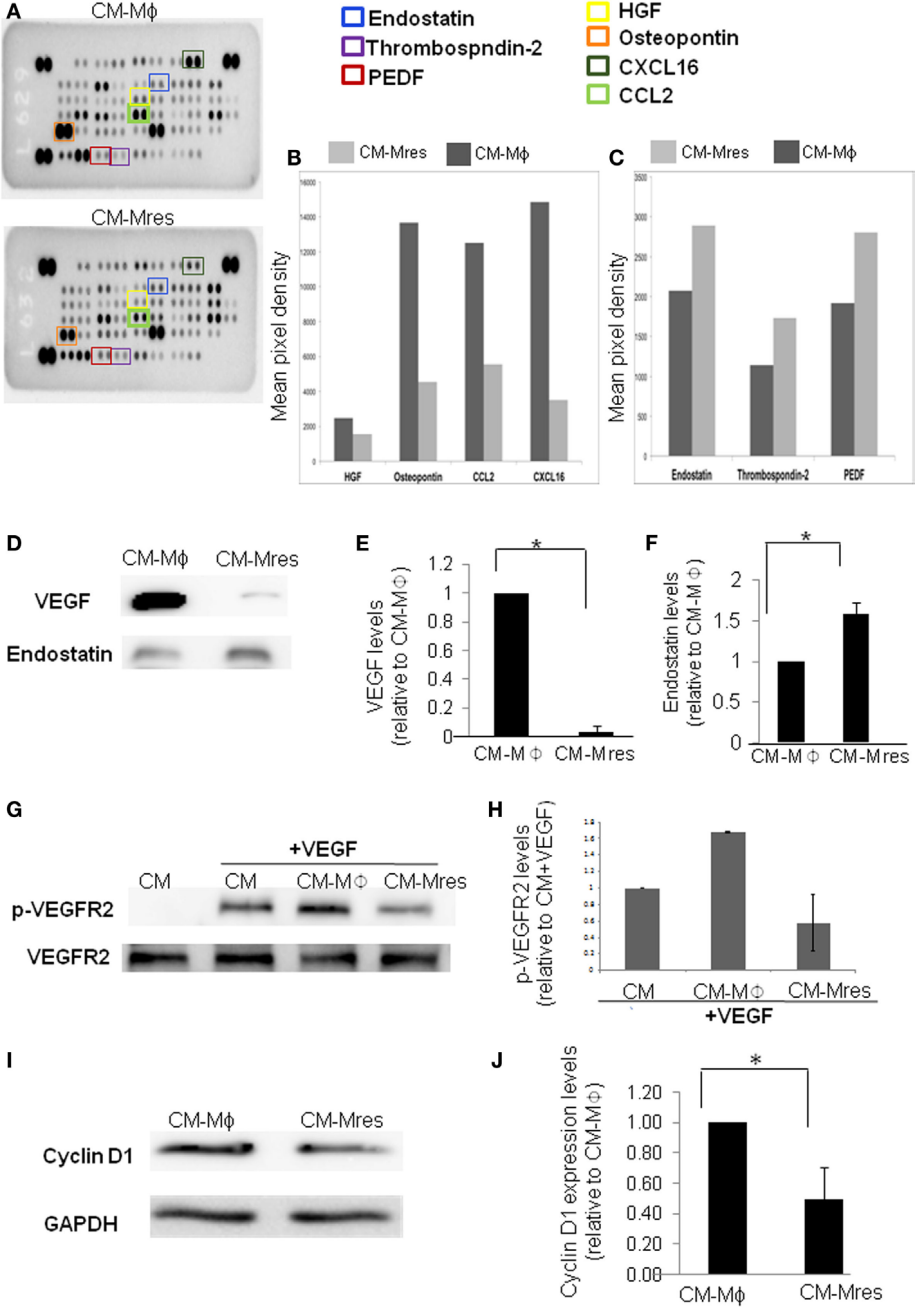
Antiangiogenic factors levels are higher in CM-Mres compared with CM-Mϕ. **(A)** The Proteome Profiler Mouse Angiogenesis Array Kit (Catalog # ARY015) was used to simultaneously assess the relative levels of 53 mouse angiogenesis-related proteins in either CM-Mϕ (upper panel) or CM-Mres (lower panel). **(B)** Quantification of pro-angiogenic factors; HGF, osteopontin, CXCL16, and CCL2. **(C)** Quantification of antiangiogenic factors; endostatin, thrombospondin-2, and PEDF. The histograms **(B,C)** were generated by quantifying the mean spot pixel density from the arrays using image software analysis. **(D)** Representative western blot analysis of vascular endothelial growth factor (VEGF) and endostatin levels in CM-Mϕ compared with CM-Mres. **(E,F)** Quantification of VEGF **(E)** and endostatin levels **(F)**. Densitometry values in panels **(E,F)** were normalized to CM-Mϕ. *n* = 3, *t*-test, *p < 0.05. **(G)** Representative western blot of VEGFR2 phosphorylation in human umbilical vein endothelial cell (HUVEC) induced by VEGF (25 ng/ml) in the presence of the different conditioned media and its quantification **(H)**. **(H)** Densitometry values of p-VEGFR2 were normalized to treatment with condition medium (CM) + VEGF (*n* = 2). Columns; mean, bars; STD. **(I)** Representative western blot of cyclin D1 expression levels in HUVEC treated with either CM-Mϕ or CM-Mres and its quantification **(J)**. **(J)** Densitometry values were normalized to treatment with CM-Mϕ. Columns; mean, bars; STD, *n* = 3, *t*-test, *p < 0.05.

Angiogenesis is regulated by a balance between angiogenic and antiangiogenic factors. Given that CM-Mres inhibited angiogenesis *in vitro, ex vivo*, and *in vivo*, has promoted us to determine the presence and predominance of antiangiogenic mediator/s over angiogenic factors in CM-Mres. To this end, mouse angiogenesis array was utilized to detect pro- and antiangiogenic factors in CM-Mres compared with CM-Mϕ (Figures 5A–C). Initial dot blot analysis revealed several pro-angiogenic factors with lower levels in CM-Mres compared with CM-Mϕ such as osteopoetin (21), HGF (22), CXCL16 (23), and CCL2 (24) (Figure 5B). Whereas, increase in the levels of the antiangiogenic factors endostatin, PEDF and thrombospondin-2 [reviewed in Nyberg et al. (25)] was observed in CM-Mres compared with their levels in CM-Mϕ (Figure 5C). Notably, the levels of the central pro-angiogenic mediator VEGF (26) decreased significantly in CM-Mres compared with its levels in CM-Mϕ (determined by western blot analysis; Figures 5D,E), whereas the levels of endostatin; an inhibitor of VEGF mediated signaling (25), was significantly higher in CM-Mres compared with CM-Mϕ (determined by western blot analysis; Figures 5D,F). Next, we tested VEGFR2 phosphorylation, given that endostatin was previously shown to block VEGF-induced tyrosine phosphorylation of VEGFR2 in HUVEC (see Materials and Methods) (27). Indeed, VEGFR2 phosphorylation on Y195 was reduced upon treatment with CM-Mres compared with treatment with CM-Mϕ (Figures 5G–H). Furthermore, CM-Mres inhibited significantly Cyclin D1 expression in HUVEC compared with treatment with CM-Mϕ (determined by western blot analysis; Figures 5I,J). These results are in accordance with previous studies demonstrating downregulation of Cyclin D1 expression upon endostatin treatment (28). Overall, our results suggest that reduction in the levels of VEGF and increase in endostatin levels in CM-Mres may mediate in part the antiangiogenic effect of CM-Mres by inhibiting VEGFR2 mediated signaling.

## Discussion

Macrophages play a fundamental role in wound healing by generating bioactive mediators that stimulate angiogenesis and fibroplasia (29, 30). However, the potential role of macrophages in resolving tissue repair by inhibiting angiogenesis is largely unknown (9).

Angiogenesis is a multifaceted process required to facilitate restoration of the damaged tissue during wound healing. This process is orchestrated by: (1) remodeling of the extracellular matrix, (2) proliferation and migration/chemotaxis of endothelial cells, and (3) assembly of endothelial cells to vessel tube and its stabilization by pericytes and smooth muscle cells (17). In this study, we demonstrate for the first time to our knowledge that mediators generated by pro-resolving CD11b^low^ macrophages (Mres) that participate during resolution of acute murine peritonitis (11) inhibit angiogenesis *in vitro* and *in vivo*. The multiple parameters of angiogenesis, proliferation, viability, and motility, were modulated by CM-Mres thus culminating in overall significant and robust inhibition of the angiogenic process.

*In vitro*, we demonstrated that bioactive mediators produced by Mres inhibited HUVEC proliferation significantly and enhanced their apoptosis. This was depicted by increase in the percentage of cells positive for TUNEL and increase in caspase 3/7 activity. Furthermore, a significant reduction in the migration capacity of the cells was observed upon exposing HUVEC to CM-Mres compared with treatment with CM-Mϕ as determined by the wound migration assay and time-lapse live video microscopy. Furthermore, tubular formation of HUVEC on BME was also significantly reduced upon exposure to CM-Mres in comparison to treatment with CM-Mϕ. This was further supported by a significant reduction in the number of bifurcations in the tubular network formed by the endothelial cells treated with CM-Mres in comparison to treatment with CM-Mϕ. Similarly, CM-Mres restrained vascular outgrowth, whereas CM-Mϕ promoted vascular outgrowth, in the rat aorta ring and CAM model systems. Notably, rat aorta ring assay and CAM assay exhibit multiple cell processes involved in angiogenesis as depicted *in vitro* and allow analysis of angiogenesis in an environment composed of multiple cell types and in the physiological context, respectively (31–33). Hence, our results demonstrate that bioactive mediators generated by Mres can inhibit different stages in the angiogenesis process.

Inhibition of angiogenesis is dependent on tilting the delicate balance between pro- and antiangiogenic factors. Therefore, if antiangiogenic factors predominate the angiogenic factors then angiogenesis will not occur (17, 34). Initial insight into the balance between pro- and antiangiogenic factors in CM-Mres vs. CM-Mϕ was obtained by performing a mouse angiogenesis array. Preliminary results demonstrate reduction in some of the pro-angiogenic growth factors and chemokines such as HGF (22), osteopontin (21), CCL2 (35), and CXCL16 (36) in CM-Mres compared with CM-Mϕ. Notably, these mediators were shown previously to modulate motility proliferation and/or survival of endothelial cells. Furthermore, an increase in some of the antiangiogenic mediators such as PEDF, thrombospondin-2 and endostatin [reviewed in Nyberg et al. (25)] was observed in CM-Mres in comparison to CM-Mϕ. Endostatin is a potent inhibitor of angiogenesis. It is a 20-kDa proteolytic fragment of collagen XVIII, which exerts its antiangiogenic effect by inhibiting VEGF binding to VEGFR2 and thus prevents VEGFR2 phosphorylation and activation (27). Notably, along with the increase in endostatin levels in CM-Mres, VEGF levels were significantly reduced in comparison to CM-Mϕ. Hence, this tilt in the balance between VEGF and endostatin in CM-Mres may have resulted in the antiangiogenic effect of the CM-Mres on HUVEC. To further explore this, we tested whether CM-Mres can inhibit VEGF-induced tyrosine phosphorylation of VEGFR2 in HUVEC (27). Indeed, VEGFR2 phosphorylation on Y195 was reduced upon treatment with CM-Mres compared with treatment with CM-Mϕ. Furthermore, we demonstrated reduction in cyclin D1 expression in HUVEC treated with CM-Mres compared with treatment with CM-Mϕ. Notably, in these experiments HUVECs were exposed to angiogenic factors that were added, for instance, bFGF (supplemented in all prepared conditioned media) or present in the CM-Mϕ, such as VEGF. This is in line with a previous report demonstrating endostatin-induced downregulation of cyclin D1 (28) resulting in G1 arrest of endothelial cells that were either treated with bFGF or VEGF. Similarly, the reduction in motility and increase in caspase 3 activity upon treatment with CM-Mres can also be attributed to endostatin angiostatic activity, as described previously (37). Overall, our results suggest that reduction in the levels of VEGF and increase in endostatin levels in CM-Mres may mediate in part the antiangiogenic effect of CM-Mres by inhibiting VEGFR2-mediated downstream signaling. VEGFR2 activation and induction of its downstream signaling by VEGF is one of the key pathways in the angiogenesis process and wound healing repair (38, 39). Hence, the potential antiangiogenic activity of CM-Mres *via* inhibition of VEGFR2 downstream signaling warrants further future experimentation. Overall, our results suggest that pro-resolving CD11b^low^ macrophages can resolve tissue repair by secreting angiostatic mediators, such as endostatin, thus insuring tissue restoration to its homeostatic state.

## Ethics Statement

This study was carried out in accordance with the recommendations of University of Haifa Animal Ethics Committee guidelines. The protocol was approved by the University of Haifa Animal Ethics Committee.

## Author Contributions

SM, VD, KW, and OGranski performed the experiments and analyzed the data. SM, VD, and KW prepared the figures. SS and OGilon prepared the conditioned media of the macrophages and performed FACS analysis. AM and IS performed the aorta ring assay and its analysis. IS reviewed the manuscript. DB conceived the project, designed the experiments, analyzed the data, and wrote the manuscript.

## Conflict of Interest Statement

The authors declare that the research was conducted in the absence of any commercial or financial relationships that could be construed as a potential conflict of interest. The handling Editor declared a shared affiliation, though no other collaboration, with the authors.

## Supplementary Material

Video S1 Time lapse video microscopy of HUVEC treated with CM-Mres.

Video S2 Time lapse video microscopy of HUVEC treated with CM-Mϕ.
