# Supplementary material for: Soluble Mediators Produced by Pro-Resolving Macrophages Inhibit Angiogenesis
Source: Front Immunol. 2018 Apr 25;9:768. doi: 10.3389/fimmu.2018.00768 (PMC5996919; doi:10.3389/fimmu.2018.00768)
Supplement: Supplementary file 3 [file Presentation_1.pptx]

## Slide 1
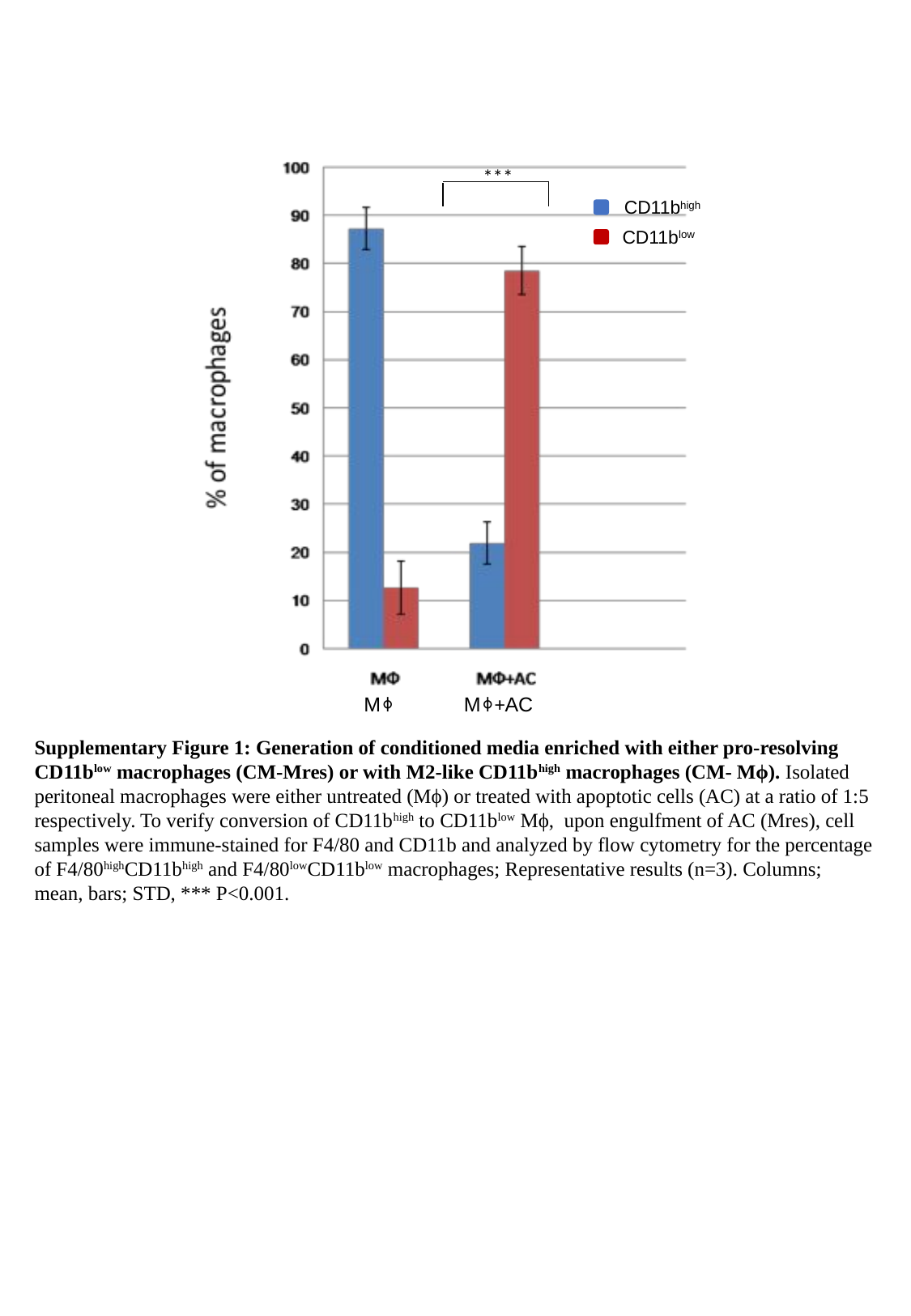

***
CD11bhigh
CD11blow
M⏀
M⏀+AC
Supplementary Figure 1: Generation of conditioned media enriched with either pro-resolving CD11blow macrophages (CM-Mres) or with M2-like CD11bhigh macrophages (CM- Mϕ). Isolated peritoneal macrophages were either untreated (Mϕ) or treated with apoptotic cells (AC) at a ratio of 1:5 respectively. To verify conversion of CD11bhigh to CD11blow Mϕ, upon engulfment of AC (Mres), cell samples were immune-stained for F4/80 and CD11b and analyzed by flow cytometry for the percentage of F4/80highCD11bhigh and F4/80lowCD11blow macrophages; Representative results (n=3). Columns; mean, bars; STD, *** P<0.001.

## Slide 2
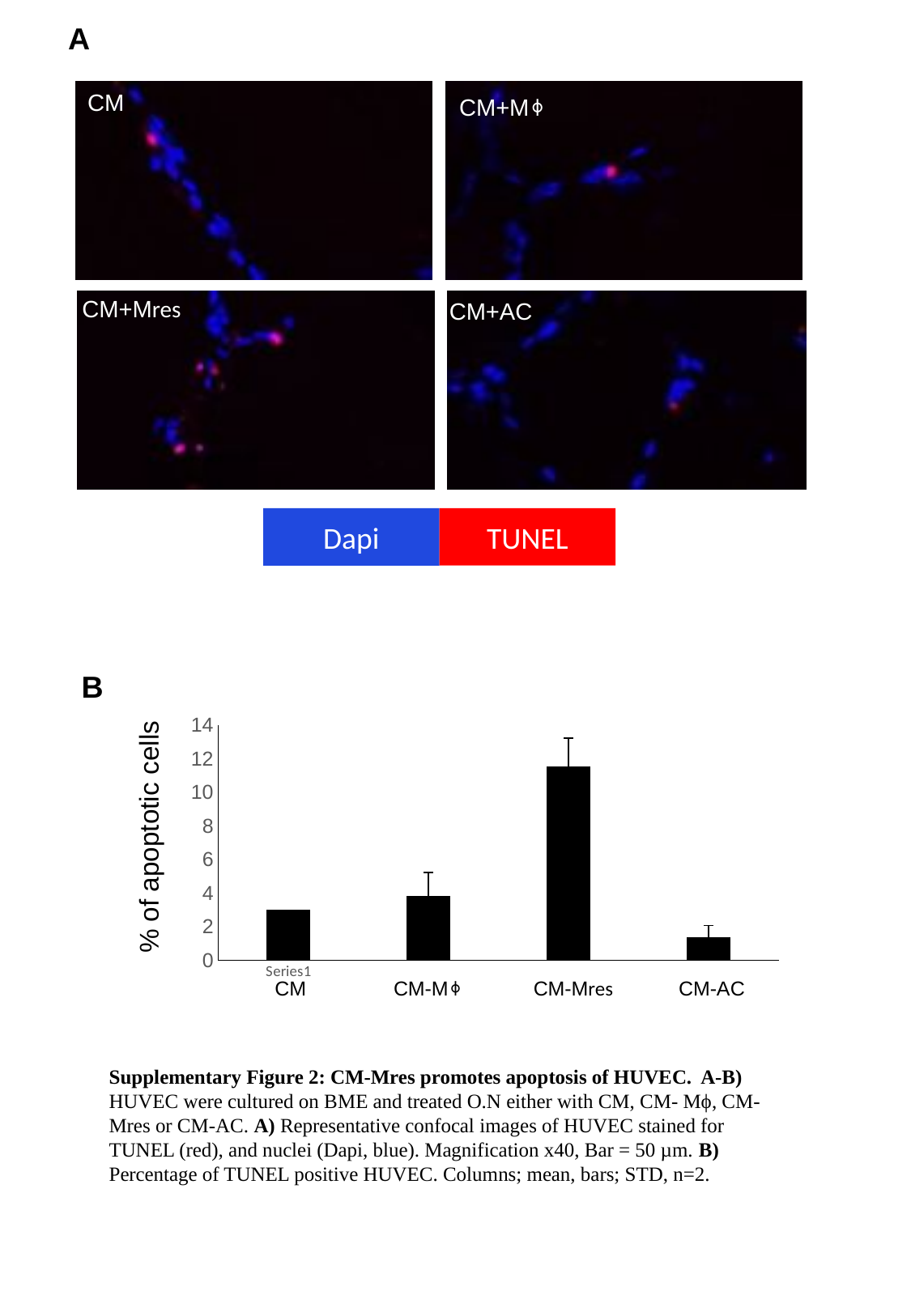

A
CM
CM+M⏀
CM+Mres
CM+AC
TUNEL
Dapi
B
### Chart
| Category | average |
|---|---|
| | 3.0 |
| | 3.8365034399999853 |
| | 11.545 |
| | 1.3800000000000001 |% of apoptotic cells
CM
CM-M⏀
CM-Mres
CM-AC
Supplementary Figure 2: CM-Mres promotes apoptosis of HUVEC. A-B) HUVEC were cultured on BME and treated O.N either with CM, CM- Mϕ, CM-Mres or CM-AC. A) Representative confocal images of HUVEC stained for TUNEL (red), and nuclei (Dapi, blue). Magnification x40, Bar = 50 µm. B) Percentage of TUNEL positive HUVEC. Columns; mean, bars; STD, n=2.

## Slide 3
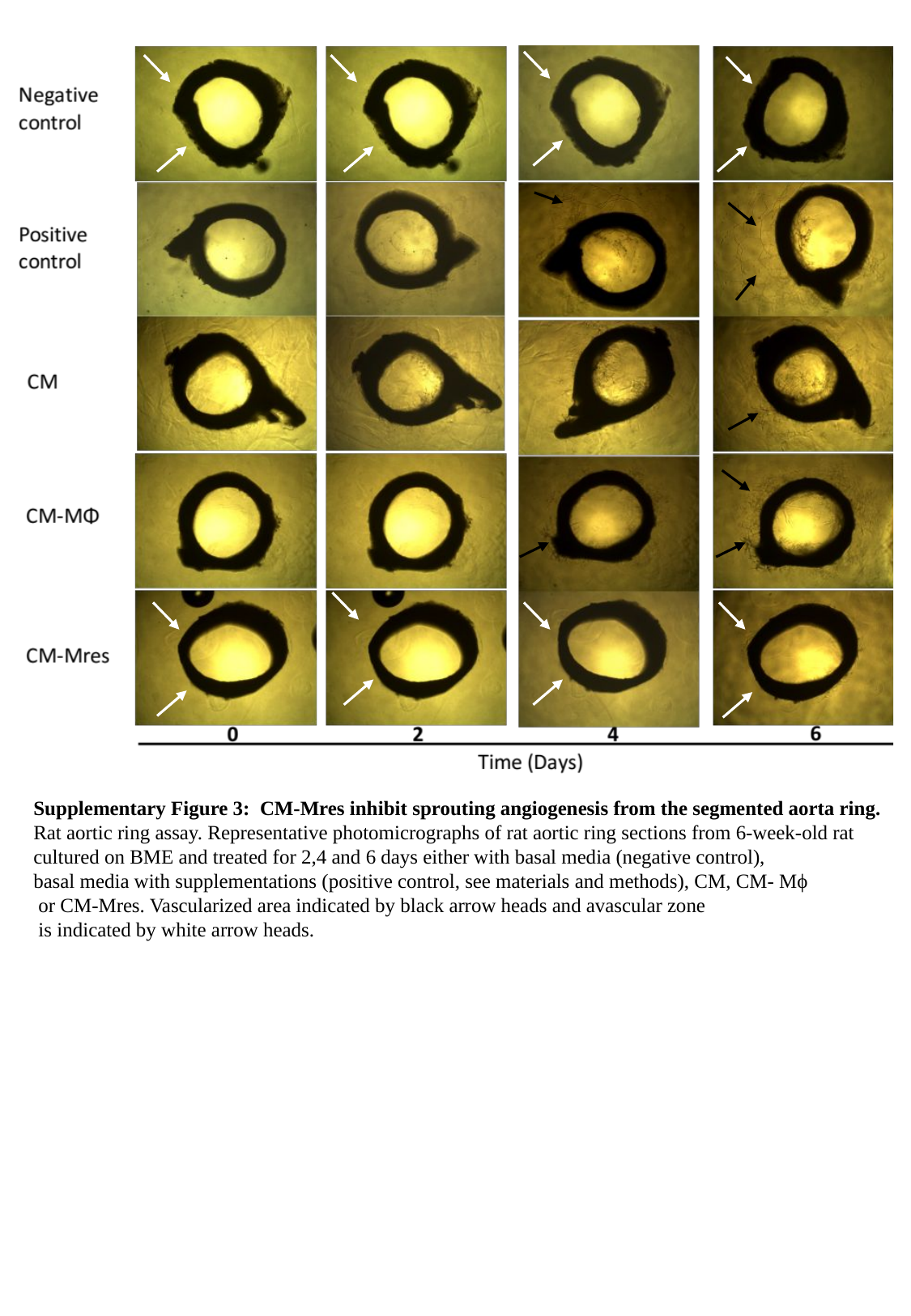

Supplementary Figure 3: CM-Mres inhibit sprouting angiogenesis from the segmented aorta ring.
Rat aortic ring assay. Representative photomicrographs of rat aortic ring sections from 6-week-old rat
cultured on BME and treated for 2,4 and 6 days either with basal media (negative control),
basal media with supplementations (positive control, see materials and methods), CM, CM- Mϕ
 or CM-Mres. Vascularized area indicated by black arrow heads and avascular zone
 is indicated by white arrow heads.
