# Supplementary material for: Soluble Mediators Produced by Pro-Resolving Macrophages Inhibit Angiogenesis
Source: Front Immunol. 2018 Apr 25;9:768. doi: 10.3389/fimmu.2018.00768 (PMC5996919; doi:10.3389/fimmu.2018.00768)
Supplement: Supplementary file 4 [file Table_1.PDF]

Component	Final concentration
rh VEGF	5 ng/ml
rh EGF	5 ng/ml
rh FGF basic	5 ng/ml
rh IGF1	15 ng/ml
L-glutamine	10 mM
Heparin sulfate	0.75 Units/ml
Hydrocortisone hemisuccinate	1 µg/ml
Fetal Bovine Serum	2%
Ascorbic acid	50 µg/ml

Supplementary Table 1: Supplementations for vascular cell basal medium for aorta ring assay.
